# Construction and Optimization of an Ecological Security Pattern Based on the MCR Model: A Case Study of the Minjiang River Basin in Eastern China

**DOI:** 10.3390/ijerph19148370

**Published:** 2022-07-08

**Authors:** Xinke Wang, Xiangqun Xie, Zhenfeng Wang, Hong Lin, Yan Liu, Huili Xie, Xingzhao Liu

**Affiliations:** 1 College Landscape Architecture, Fujian Agriculture and Forestry University, Fuzhou 350002, China; wang_xin_ke@163.com (X.W.); xiangqxie@hotmail.com (X.X.); zfwangone@hotmail.com (Z.W.); redlin15@hotmail.com (H.L.); lllnnny@163.com (Y.L.); huili_x@126.com (H.X.); 2 Innovation Center of Engineering Technology for Monitoring and Restoration of Ecological Fragile Areas in Southeast China, Ministry of Natural Resources, Fuzhou 350013, China

**Keywords:** mountain–river–forest–farmland–lake–grass, Minjiang River Basin, ecological environment assessment, MCR model, ecological security pattern, spatial analysis

## Abstract

The Minjiang River Basin is one of the first pilot areas for ecological conservation and the restoration of mountain–river–forest–farmland–lake–grass in China. Taking the Minjiang River Basin as an example, this paper selected the importance of ecosystem service functions and ecological sensitivity to evaluate the ecological environment and identify ecological sources. Furthermore, we constructed an ecological resistance surface using artificial and natural interference factors. Through a minimum cumulative resistance model (MCR), the ecological security pattern (ESP) of “two barriers, one belt, many corridors, and many spots” was constructed. Research shows that: (1) In total, 43 ecological sources were identified, with a total area of 523 km^2^, accounting for 0.6% of the total land area. These were mainly distributed in the southwest and northwest of the Minjiang River Basin, such as in Zhangping, covered forest land, and cultivated land. (2) The connectivity of the network was low, and the spatial distribution of the ecological pinch points was uneven. A total of 118 ecological corridors and 22 important ecological pinch points were identified. The total length of the ecological corridor is 3,732,051.88 km, which is dense on the left side and sparse on the right side. (3) The ecological restoration area was composed of a low ecological safety area and a lower ecological safety area; the ecological control area was composed of a medium ecological safety area and a higher ecological safety area; and the ecological conservation area was composed of a high ecological safety area, at 6.5%, 27.7%, and 65.8%, respectively. Constructing the ESP of the Minjiang River Basin is important for promoting harmonious socioeconomic development and ecological protection. In addition, it can provide a reference basis for other experimental areas of mountain–river–forest–farmland–lake–grass.

## 1. Introduction

The increasingly intense human activities have brought about problems such as habitat fragmentation, loss of biodiversity, and a decline in ecosystem services, which seriously restrict the sustainable development of the region [1,2,3,4]. The connectivity of the ecological security network has been destroyed to a certain extent [5]. Since the 1970s, many scholars have researched ecological environmental protection [6,7]. In the 1990s, theories and methods related to ESP were put forward [8]. Constructing a pattern of ecological security (ESP) helps to maintain the integrity of the process of an ecosystem structure and improve the connectivity of different habitats through the “point–line–plane” model; in addition, this can effectively help to avoid the lasting negative influence of habitat fragmentation on the ecological environment [9], conducive to optimizing the ecological security pattern and promoting the construction of ecological civilization [10,11,12].

Today, “identifying ecological sources, constructing ecological resistance surfaces, and extracting ecological corridors and nodes “has gradually become the basic paradigm of ecological security pattern research [13,14,15]. Among them, the ecological source is the key ecological patch, which can not only promote the ecological process, but also maintain the integrity and stability of the ecosystem [16], and play an important role in maintaining regional ecological security [17]. An ecological source is a large habitat plate with important ecological functions and strong sensitivity, being the source and sink of the ecosystem services supply [18]. The identification of ecological sources mainly adopts two methods: qualitative evaluation and quantitative evaluation of ecosystem structure [19]. According to the natural background conditions and the characteristics of habitat patches in the study area, relevant scholars directly regard important ecological land, such as nature reserves, forest parks, and wetland parks, as ecological sources [8,20]. This approach largely ignores intrinsic differences. Therefore, relevant scholars have tried to construct a quantitative comprehensive evaluation index to avoid this problem [21]. This study also considers this situation, and selects 6 comprehensive evaluation indicators to evaluate the ecological environment, so as to identify the ecological sources. The ecological resistance surface reflects the resistance to species migration and energy flow, which have an important impact on species diversity and complexity [22]. Usually, land use type is the main factor combined with other factors to construct the resistance surface [23,24,25]. Ecological corridors are the structural basis for species migration and energy flow in ecological networks [26,27]. Represents possible areas for organisms to move from one ecological source to another [28]. Ecological corridors play an important role in strengthening links between ecological sources [12]. The minimum cumulative resistance (MCR) model is currently an important method used by scholars to extract ecological corridors [1], which is based on the theory of “source-sink”. It can better reflect the impact of landscape pattern changes on the evolution of ecological processes [1].

The Minjiang River Basin is one of the first pilot areas for ecological conservation and the restoration of mountain–river–forest–farmland–lake–grass in China. It is also an extremely important area for forest production and economic development in Fujian Province in China [29]. Some documents show that as of 2017, the water quality in the Minjiang River Basin that cannot meet the requirements of domestic water still exists, mainly distributed in the middle and lower reaches of the main stream of the Minjiang River in the Dazhangxi River Basin [30] and Minjiang Estuary [31]. Soil and water loss is still serious. According to the National Soil and Water Conservation Plan (2015–2030), there are 22 key counties for soil and water loss control in Fujian Province, of which the Minjiang River Basin accounts for 11 counties [32]. Forests and biodiversity are under threat of degradation [32].

Given this situation, it is of great significance to study the ecological environment of the Minjiang River Basin comprehensively, identify the specific areas facing threats in the basin, construct its ESP, and propose optimization strategies. This study follows the principles of adapting measures to local conditions and data availability. To identify the areas in the Minjiang River Basin that are threatened by soil erosion, biodiversity degradation, etc. The functional importance and functional importance of biodiversity conservation have been comprehensively evaluated for its ecological environment. According to the comprehensive evaluation results, identifying its ecological source, extracting its ecological corridors and ecological nodes, analyzing its vulnerable and sensitive areas, and proposing an ecological restoration plan accordingly, it can lay a foundation for the protection and restoration of mountain–river–forest–farmland–lake–grass, and provide a replicable experience for the construction of similar projects in Fujian province and throughout China.

## 2. Study Area and Data Sources

### 2.1. Overview of the Study Area

**Figure 1:**
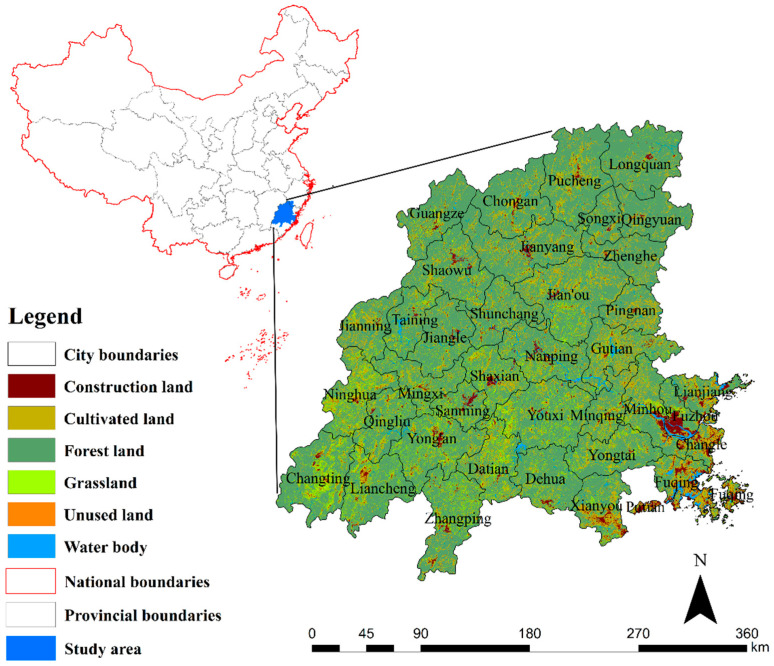
Geographical location and land use of the Minjiang River Basin.

The Minjiang River Basin (116°23′–119°35′ E, 25°23′–28°16′ N) originates from the Wuyishan and Xianxia Mountains [29], with a total length of 6207 km and a basin area of 65,623.95 km^2^, accounting for about half of the area of Fujian Province [33]. It flows through parts of Sanming, Nanping, Ningde, Fuzhou, Quanzhou, and Putian (Figure 1) [29]; the watershed is defined as upstream, midstream, and downstream, bounded by Nanping and a water gap. The topography of the Minjiang River Basin is high in the northwest and low in the southeast; the climate is a subtropical monsoon climate, the annual precipitation is about 1700 mm, and the main meteorological disasters are rainstorms, flooding, and typhoons [32]. The soil types are sandy loam, loamy sandy soil, clay loam, silty loam, and so on.

### 2.2. Data Sources

Land-use data with an accuracy of 30 m from Globeland30 in 2020 and soil data with an accuracy of 1:1 million from the soil science data of the National Earth System Science Data Sharing Service Platform were used [34]. The DEM data have an accuracy of 30 m and are derived from the geospatial data cloud (https://www.gscloud.cn, accessed on 11 April 2021). The basin’s rainfall and other meteorological data were derived from the China Meteorological Data Network (https://data.cma.cn, accessed on 11 April 2021), the resolution is 1000 m. Net primary productivity (NPP) data were derived from NASA MODIS satellite products (https://modis.gsfc.nasa.gov/, accessed on 11 April 2021), the resolution is 1000 m. The basic geographic data and spatial administrative boundary vector data came from the National Basic Geographic Information System database (http://www.ngcc.cn/ngcc/, accessed on 11 April 2021). Land use and vegetation cover data came from the Chinese Academy of Sciences (http://www.resdc.cn/, accessed on 11 April 2021).

## 3. Methods

First, given the ecological problems in the Minjiang River Basin, for the ecological sensitivity assessment, we selected three indicators: sensitivity to soil and water loss (*SSWL*), sensitivity to soil erosion (*SSE*), and sensitivity to land desertification (*SLD*). To assess the importance of ecosystem functions, we selected three indicators: water conservation function (*WR*), soil and water conservation function (*S_pro_*), and biodiversity maintenance function (*S_bio_*). In this paper, the weight of evaluation factors was determined by AHP, and the comparison of the importance of evaluation factors was made by referring to previous literature [35] and consulting experts to weaken the subjectivity of this method. The weights of *SSWL*, *SSE,* and *SLD* were 0.43, 0.39, and 0.18, respectively, and the weights of *WR, S_pro,_* and *S_bio_* were 0.45, 0.30, and 0.25, respectively. The integrated ecological sensitivity and importance of the integrated ecosystem service functions were obtained using a grid calculator; then, the integrated ecological environment assessment was obtained by spatial superposition. It was divided into five grades by the natural discontinuity method: generally important, relatively important, medium important, highly important, and extremely important. The most important patches with a distance of less than 500 m were aggregated into relatively intact patches, and patches with a distance greater than 8 km^2^ were selected as the ecological source.

Second, land use intensity, vegetation coverage, and slope were selected as natural disturbance factors, and the distance from highways and county roads was selected as a human disturbance factor to construct the minimum cumulative resistance surface; then, the ecological corridor and ecological nodes were extracted with the MCR model.

**Figure 2:**
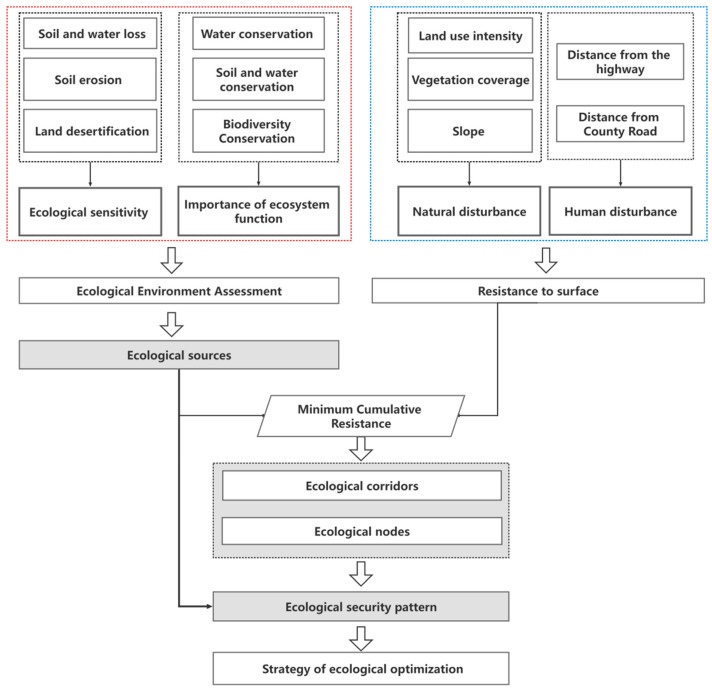
The methodological framework of the study.

Finally, the ecological security pattern was constructed, the security level was divided based on the minimum cumulative resistance surface, and a corresponding optimization strategy was proposed (Figure 2).

### 3.1. Analytic Hierarchy Process (AHP) Method

The multi-criteria decision-making (MCDM) technique is effective for building comprehensive evaluation models, among which AHP is one of the most widely used methods [36]. In this study, AHP was used to determine the weights of the evaluation indicators in the comprehensive evaluation factors. The first step in AHP is to construct a hierarchy that includes objective, criterion, and solution levels [37]. Subsequently, a pairwise priority comparison of elements at each level needs to be made based on prior knowledge, where Saaty’s 1–9 scale method can be used [38]. Thus far, most core pairwise comparison matrices in the AHP method have been established, and their consistency needs to be checked using the following formula:

$$CR=\frac{CI}{RI}\;$$

$$CI=\frac{\lambda_{max}}{n-1}-\frac{n}{n-1}$$

where *λ_max_* is the largest eigenvalue of the pairwise comparison matrix, and n is the order of the matrix. There is a corresponding relationship between the random index (*RI*) and n; that is, when *n* is 1–10, *RI* is 0, 0, 0.58, 0.90, 1.12, 1.24, 1.32, 1.41, 1.45, and 1.49, respectively. If *CR* < 0.1, the weight distribution is sensible [39].

### 3.2. Eco-Environmental Assessment and Identification of Ecological Source Areas

Eco-environmental assessment refers to the comprehensive impact of the quality of service functions provided by ecosystems and the sensitivity of ecosystems to external disturbances. On this basis, we considered patches with high ecological value as alternative areas for ecological sources [40].

#### 3.2.1. Selection of Evaluation Factors and Grade Division

Ecological sensitivity refers to the sensitivity of an ecosystem to sudden environmental changes and human activities, which indicates the possibility of an ecological imbalance [41]. It is closely related to ecological protection and the restoration of mountain–river–forest-farmland–lake–grass. The ecosystem service function refers to the beneficial effect that the ecosystem and species depend on to maintain and realize the environmental condition of human existence [42], including water resource protection, soil, and water conservation, and biodiversity protection [40]. Evaluate ecosystem function importance and analyze the law of the regional differentiation of ecosystem service functions, which can clarify the important regions of ecosystem service [43].

Considering that the ecological environment of the study area mainly has problems such as water pollution, soil erosion, and biodiversity degradation, in order to find out the ecological background problems and better guide the government to optimize the ecological environment. This paper selected *SSWL*, *SSE*, and *SLD* to evaluate the ecological sensitivity of the Minjiang River Basin, and *WR*, *S_pro_*, and *S_bio_* were selected to evaluate the importance of ecosystem functions in the Minjiang River Basin.

Evaluating *SSWL* can determine the area susceptible to soil and water loss and evaluate the sensitivity of the ecosystem to human activities [44]. The formula is as follows:

$$[SSWL]=\sqrt[{4}]{R\times K\times LS\times C}$$

Rainfall erodibility factor (*R*):

$$R=\overset{n}{\underset{i=1}{\sum }}\left(-2.6398+0.3046P_{i}\right)$$

The formula is based on the “Technical guidelines for the assessment of suitability for resource carrying capacity and territorial and spatial development”. $P_{i}$ is the average monthly rainfall for many years. Monthly rainfall data from 1990 to 2015 were obtained from the Chinese Academy of Sciences Resource Environment Data Center. After calculating the monthly average rainfall, a grid layer of rainfall erosivity was created using spatial interpolation with a resolution of 1000 m.

The soil erodibility factor (*K*) was calculated from 1:1 million soil maps. It was released by the Soil Science Data Center of the National Earth System Science Data Sharing Service platform. Soil texture classification was based on international system classification standards. 

The topographic relief factor (*LS*) reflects the comprehensive characteristics of slope and length, and is one of the main features of topography and landforms. This refers to the difference between the highest altitude and the lowest altitude in a specific area. The topographic relief was obtained with focal statistics in the 36*36 neighborhood analysis on the ArcGIS platform.

Vegetation coverage (*C*) was calculated from the 30-m precision land-use map and resampled to 1000 m resolution. Table 1 shows the classification criteria for each factor.

The sensitivity of soil erosion (*SSE*) is a comprehensive evaluation based on the general soil erosion equation [45], which comprehensively considers the ecological environment factors of the Minjiang River Basin and uses GIS technology. *W_i_* is the sensitivity level value of the *i* factor. In this study, four factors were selected to evaluate soil erosion sensitivity: slope length (*LS*), rainfall erodibility (*R*), soil texture (*K*), and vegetation coverage (*C*). The formula is as follows:

$$[SSE]=\frac{1}{n}\sum_{i=1}^{n}W_{i}$$

The sensitivity of land desertification (*SLD*) was evaluated by GIS technology, referred to the “Interim Regulations for Ecological Function Zoning”, considering the factors of the dryness index (*I*), the days of sand blowing (*W*), soil texture (*K*) and vegetation coverage (*C*) [35]. The aridity index was derived from the spatial interpolation of the mean aridity index for 2010–2015. The drought index came from the Fujian Meteorological Bureau. The sandstorm days were obtained from the “Daily Value data set of China surface climate data (V3.0)” of the China Meteorological Data Network. In this paper, the average sandstorm days from 2010 to 2015 were selected to calculate the sandstorm days in the study area; they were then used to generate a raster image using spatial interpolation. The formula is as follows:

$$[SLD]=\sqrt[{4}]{I\times W\times K\times C}$$

The functions of water source conservation (*WR*) include regulating water flow, achieving water circulation, and ensuring water quality [37]. The evaluation of the importance of water conservation functions is carried out according to the quantitative index method stipulated in the “National Ecological Protection Red Line—Technical Guidelines for the Delineation of Ecological Function Red Lines (Trial)” [35]. The calculation formula is as follows: *WR* is the ecosystem water conservation capacity index, *NPP_mean_* is the annual average of vegetation net primary productivity (2005–2010), and *F_sic_* is the soil percolation factor, which was obtained by dividing the T_USDA_TEX in the global soil data (HWSD) by 13. According to the soil texture classification of the United States Department of Agriculture (USDA), the 13 soil texture types are equally assigned between 0 and 1, such as clay (heavy) is 1/13, silty clay is 2/13, …, sand is 1 [35]. *F_pre_* was calculated by averaging rainfall over the period 1990–2015, and *F_sio_* is the slope factor.

$$WR=NPP_{mean}\times F_{sic}\times F_{pre}\times (1-F_{sio})$$

The function of the evaluation of the soil and water conservation is to calculate the capacity for soil and water conservation in a specific area. Among them, *S_pro_* is the index of the soil and water conservation capacity, *K* is the soil erodibility factor, *F_sio_* is the slope factor, and *NPP_mean_* is the annual average of *NPP* (2005–2010).

$$S_{pro}=NPP_{mean}\times (1-K)\times (1-F_{sio})$$

The evaluation of biodiversity maintenance functions can determine the role of ecosystems in maintaining genetics, species, and ecosystem diversity. The formula is as follows: *S_bio_* is the Service Ability Index of Biodiversity, *F_tem_* is the annual average temperature (1990–2015), *F_pre_* is the annual average precipitation (1990–2015), and *NPP_mean_* is the annual average net primary productivity (2005–2010).

$$S_{bio}=NPP_{mean}\times F_{pre}\times F_{tem}\times (1-F_{alt})$$

#### 3.2.2. Factor Grading Assignment

In 2020, the Ministry of Natural Resources of the People’s Republic of China issued guidelines for evaluating resource and environmental carrying capacity and territorial development suitability. This study then referred to this document and used natural discontinuities to rank *R*, *K*, *LS*, *C*, *I*, and *W.* The soil texture classification was based on the triangle coordinate diagram of soil texture classification system published by the United States Department of Agriculture.

#### 3.2.3. Spatial Superposition and Source Identification

According to the ecological sensitivity and the importance of the ecosystem service function, the ecological environment of the Minjiang River Basin was comprehensively evaluated using the spatial superposition method in ArcGIS10.2 software; then, it was divided into five levels by natural discontinuities: generally important, relatively important, moderately important, highly important, and extremely important. The ecological origin is the source point of species maintenance and diffusion [18,22,46], and it is often a highly sensitive area and an important area of ecosystem function. Therefore, extremely important patches less than 500 m away from each other were clustered together, and patches larger than 8 km^2^ were chosen as ecological sources.

### 3.3. Ecological Resistance Analysis

#### 3.3.1. Minimum Cumulative Resistance Model

The minimum cumulative resistance model is the least costly path for simulating species crossing different landscape substrates from the source [47]. The resistance surface reflects the trend of species spatial movement. The basic formula is as follows:

$$MCR=f_{min}\overset{i=m}{\underset{j=n}{\sum }}D_{ij}\times R_{i}$$

In the formula, *MCR* is the minimum cumulative resistance value; *f_min_* represents the positive correlation between the minimum cumulative resistance model and the ecological process, which is an unknown monotone-increasing function [18]. ∑ represents the accumulation of the distance and resistance between grid *i* and source *j* across all elements [48]. $\;D_{ij\;}$ represents the spatial distance of ecological elements from source *j* to Landscape Unit I [18]. $R_{i}$ represents the resistance of landscape element I to the movement of an ecological element [18].

#### 3.3.2. Index System of Ecological Resistance Factors

Considering the availability of data and the principle of operability, this study selected land use intensity, vegetation coverage, and slope as natural disturbance factors; the distance from the highway and the distance from the county road were selected as the human interference factors. The closer to the road, the greater the disturbance and the greater the resistance [18]. In the literature [5,18,22], this study referred to the resistance level and the division of resistance values using an analytical hierarchy process to calculate the weight values of the resistance factors and a consistency test. Finally, the weight of each resistance factor grade was obtained, as shown in Table 2. The test coefficient, CR = 0.042 < 0.1, showed that it had good consistency (Table 3) [18].

### 3.4. Extraction of Ecological Corridors and Nodes

An ecological corridor is a spatial type of landscape ecosystem that can connect isolated and scattered ecological landscape units into a spatial distribution. Generally, it has a linear or zonal layout, which can satisfy the diffusion, migration, and transformation of species. It is an important part of constructing the integrated ecosystem of mountain–river–forest–farmland–lake–grass. First, the resistance surface of the five factors was established, and then the comprehensive resistance surface was obtained by weighted superposition. Then, the ecological corridor and ecological node area were extracted using Linkage_Mappe and Circuitscape tools in ArcGIS. The ecological nodes are located in the fragile part of the ecosystem function in the ecological corridor and play a key role in the operation of the ecological flow [49].

The construction and protection of the gradient ecological node system can make the ecological corridor more stable and enhance connectivity with the ecological source [18]. Therefore, the ecological node areas were divided into important ecological node areas and general ecological node areas according to the natural discontinuity method. In the important ecological node region, patches with a degree of connectivity greater than 1km^2^ were considered.

## 4. Results

### 4.1. Eco-Environmental Assessment and Ecological Source Identification

#### 4.1.1. Ecological Environment Assessment

**Figure 3:**
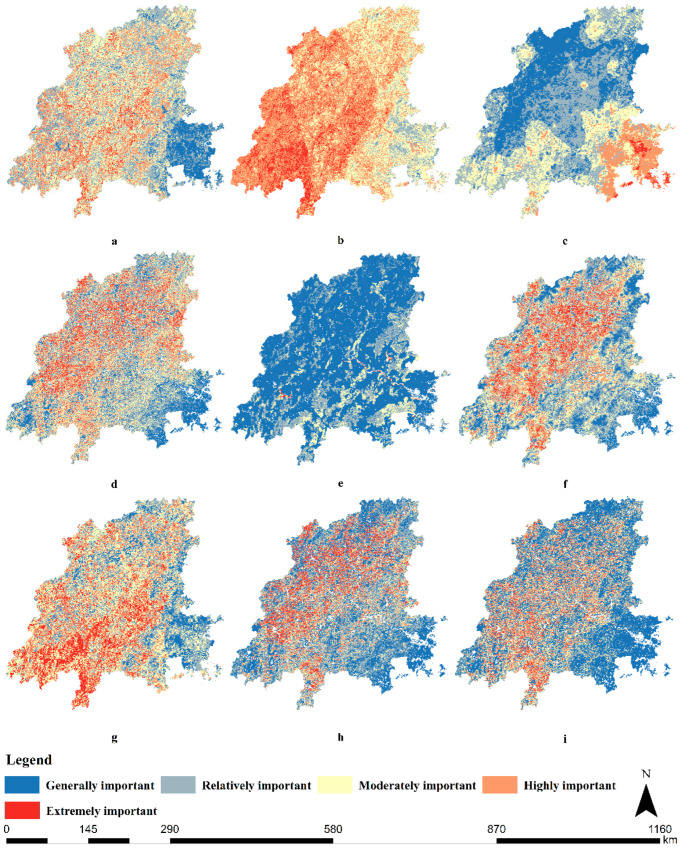
Assessment map of eco-environmental factors in the Minjiang River Basin. (**a**): Evaluation of sensitivity to soil and water loss. (**b**): Evaluation of soil erosion sensitivity. (**c**): Evaluation of sensitivity to land desertification. (**d**): Evaluation of the importance of the water conservation function. (**e**): Evaluation of the importance of the soil and water conservation function. (**f**): Evaluation of the functional importance of biodiversity. (**g**): Comprehensive assessment of ecological sensitivity. (**h**): Comprehensive assessment of the importance of ecosystem function. (**i**): Comprehensive assessment of the ecological environment.

According to the assessment results of the ecological environment factors in the Minjiang River Basin (Figure 3), the spatial distribution of *SSE*, *SSWL*, *SLD, WR*, *S_pro,_* and *S_bio_* were obviously different.

From the spatial distribution, the *SSWL* showed a circular distribution (Figure 3). The sensitivity decreased from the inner to the outer layer, and the high-sensitivity areas were mainly distributed in the upper and middle reaches of the Minjiang River Basin. The *SSE* showed a zonal distribution (Figure 3). The sensitivity decreased from west to east, and the high-sensitivity area accounted for 12.3% of the total area of the basin, which was concentrated in the upper and middle reaches of the Minjiang River Basin (Table 4). The spatial distribution of the *SLD* was patchy, with the high-sensitivity area accounting for 1.1% of the total watershed area, mainly distributed in the lower reaches of the Minjiang River Basin. The main land cover types were forest land, cultivated land, and grassland.

According to the spatial distribution of comprehensive ecological sensitivity, the upper and middle reaches of the Minjiang River Basin were more sensitive than the lower reaches. The high sensitivity and higher sensitivity areas were located near the boundary between the upper and middle reaches of the Minjiang River Basin, such as Zhangping, which accounted for 14.6% and 18.9% of the total area, respectively, mainly due to the contribution of soil erosion (Table 4). The main types of land cover were forest land, cultivated land, and grassland. The medium sensitivity areas were distributed in the upper and middle reaches of the Minjiang River Basin, covering an area of 27,001 km^2^, and accounting for 33.7% of the total watershed area (Table 4). The lower sensitivity areas and the low sensitivity areas were distributed in the eastern part of the basin and clustered in the lower reaches. This is because the upper and middle reaches of the basin are dominated by mountains and hills, and rainfall erodibility is high. Under the condition of saturated soil water, the basin is subjected to heavy rainfall and hydraulic action and is prone to ecological problems, such as soil and water slides and soil erosion [12,23]. However, the lower reaches of the basin are relatively flat, and the economy of these areas is relatively developed. Due to the strong influence of human activities, natural vegetation has been seriously destroyed, and ecological problems such as land desertification easily occur [29].

The extremely important regions in the evaluation of the *WR* were mainly distributed in the northern and western parts of the basin, accounting for 8.4% of the total area. The extremely important and highly important areas of *S_pro_* were scattered in the west and east of the basin, accounting for 0.1% and 0.2% of the total area of the basin, respectively (Table 4). The spatial distribution of *S_bio_* showed an obvious layer structure, and the extremely important and highly important areas were almost uniformly distributed in the upper reaches of the basin, accounting for 7.9% and 16.7% (Table 4).

The spatial distribution of the importance of the integrated ecosystem functions in the Minjiang River Basin was similar to that of *S_bio_* (Figure 3). Overall, the upper and middle reaches of the basin were more important than the lower reaches. This is due to the large mountainous area in the upper and middle reaches of the Minjiang River Basin, which is an important ecological function area. The extremely important, highly important, moderately important, and relatively important areas were mainly distributed in the upper and middle reaches of the Minjiang River Basin, accounting for 8.6%, 14.1%, 14.8%, and 28.8%, respectively (Table 4). One of the extremely important areas was concentrated in the middle reaches of the valley, including Zhangping. This is because Zhangping is located at the junction of Daiyun Mountain, Hawksbill Mountain, and Boping Mountain, one of the rich mineral deposits in Fujian province. In addition, Zhangping is one of the 48 key forest regions in southern China, with forest coverage of 77.9%, of which 993,000 mu are ecological noncommercial forests. Generally, important areas were distributed in the lower reaches of the river basin, accounting for 33.7% of the total area of the river basin. In general, the ecological environment of the Minjiang River Basin was characterized by “high importance in the west and low importance in the east” (Figure 3). The extremely important areas, which accounted for 4.8% of the total area of the study area, were clustered in the western part of the basin, such as Zhangping (Figure 3), and were scattered in the upper reaches of the basin, such as Wuyishan (Table 4). The highly important area accounted for 16.2% of the total watershed area and was distributed over the entire Minjiang River Basin, roughly the same as the extremely important area (Figure 3 and Table 4). The moderately important, relatively important, and generally important areas were 19.0%, 12.3%, and 47.7%, respectively, in the Minjiang River Basin (Table 4).

#### 4.1.2. Identification of Ecological Sources

As the important basis of an ecological network pattern, the ecological source region needs to consider the continuity of the landscape pattern and the ecological function of the landscape [5]. Therefore, on the basis of the comprehensive evaluation of the ecological environment, the extremely important patches with a distance of less than 500 m were aggregated into relatively intact patches, and patches larger than 8 km^2^ were selected as the ecological source. Finally, 43 ecological sources were extracted, covering an area of 523 km^2^, accounting for 0.6% of the total land area mainly distributed in the southwest and northwest of the Minjiang River Basin, such as Zhangping. In terms of ecological sources, the proportion of forest and grassland, cultivated land and construction land in the ecological source area were 81.83%, 15.87%, and 1.91%, respectively (Table 5), which shows that there is a spatial conflict between ecological protection and agricultural production; agricultural production may have some effects on ecosystem service function [50].

### 4.2. Construction of the Resistance Surface in the Minjiang River Basin

#### 4.2.1. Resistance Surface Factor

Among the land use types, forest and grassland accounted for 80.13% of the total land area, and resistance was the smallest. Water accounted for 1.41%. The proportion of cultivated land was 15.50%. The proportion of bare land was 0.11%. Construction land accounted for 2.85%, the greatest resistance. The slope reflects the topography of the Minjiang River Basin. Spatially, the eastern part of the basin is relatively flat, and the northern part is mainly distributed with hills and mountains, represented by Wuyishan. The slope of the Minjiang River Basin was divided into five grades (Table 3 and Table 6). The larger the slope, the greater the resistance. The area with a high slope (>35°) was 2465.86 km^2^, accounting for 12.29% of the total land area (Table 6). The area with a higher slope (25–35°) was 11,326.77 km^2^, accounting for 13.89% of the total land area. The area with a medium slope (15–25°) is 26,820.81 km^2^, accounting for 32.9% of the total land area. The area with a lower slope (5–15°) was 30,886.10 km^2^, accounting for 37.89% of the total land area. The area with a low slope (<5°) was 10,021.81 km^2^, accounting for 12.29% of the total land area. The slopes in the Minjiang River Basin were concentrated between 5° and 25°. The higher the vegetation coverage, the lower the resistance. In the Minjiang River Basin, 96.99% of the region had over 65% vegetation coverage; only 0.04% of the region was under 15% vegetation coverage, concentrated in the lower reaches of the Minjiang River Basin. The closer to county roads and highways, the greater the resistance, and vice versa.

#### 4.2.2. Minimum Cumulative Resistance Surface

**Figure 4:**
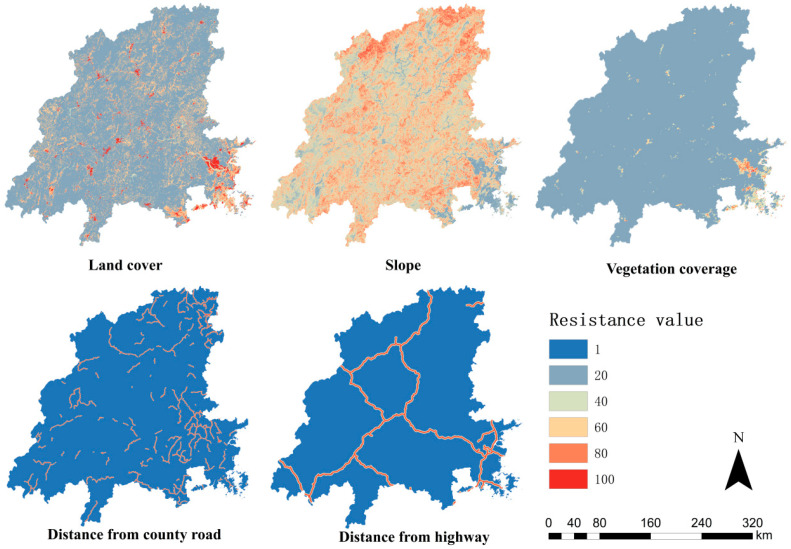
Single-factor resistance surface.

**Figure 5:**
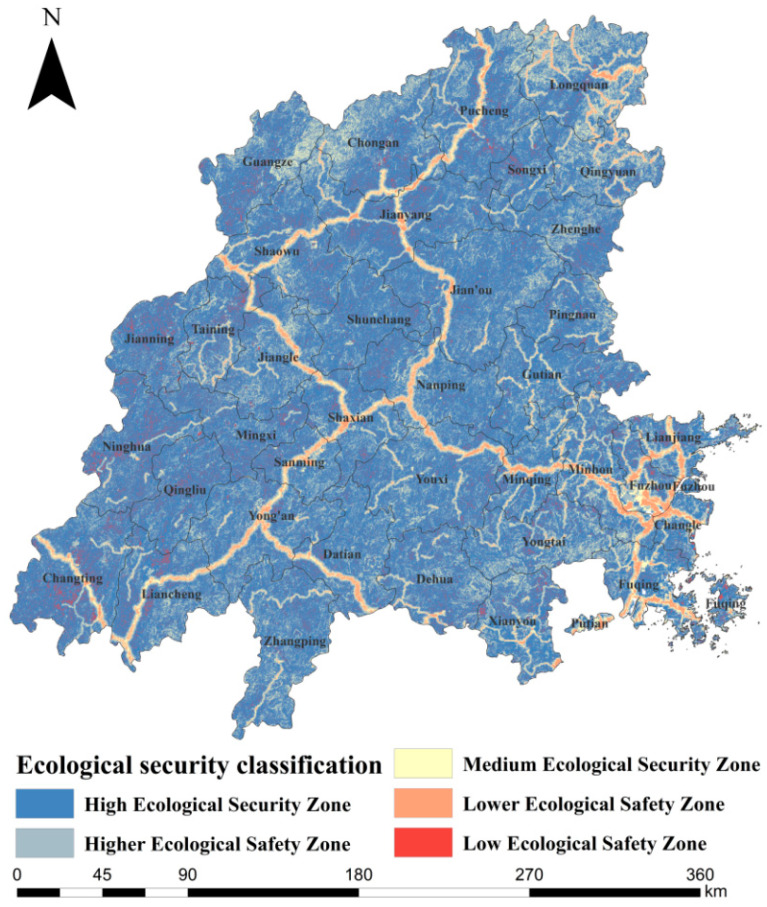
Ecological security classification of the Minjiang River Basin.

Based on the resistance factors (Figure 4), a comprehensive resistance surface was constructed for the Minjiang River Basin (Figure 5). According to the natural discontinuity method, it was divided into a high ecological safety zone, a higher ecological safety zone, a medium ecological safety zone, a lower ecological safety zone, and a low ecological safety zone. The higher the ecological resistance value, the lower the safety level. The area of the high ecological security zone was 53,288.148 km^2^, which accounted for 65.77% of the national territory area and had a uniform spatial distribution (Table 7). The area of the higher ecological safety zone was 16,858.311 km^2^, accounting for 20.81%, and the area of the medium ecological safety zone was 5608.967 km^2^, accounting for 6.92%. The area of the lower ecological safety zone was 2269.87 km^2^, accounting for 2.80%. The area of the low ecological safety zone was 3000.19 km^2^, accounting for 3.70%, which was distributed around high-speed roads.

### 4.3. Ecological Corridor and Ecological Node Identification

Based on the ecological source region and the MCR model, 118 ecological corridors were extracted, with a total length of 3,732,051.88 km. From a spatial perspective, the ecological corridor in the Minjiang River Basin has poor connectivity and low coverage, covering only the middle and upper reaches of the Minjiang River Basin. It is in the shape of a fishing net; the southwest side is dense, which connects each important ecological source well, while the northeast side ecological corridor is singular and slender, prone to ecological fracture. An ecological node is an important part of the spatial pattern of an ecological network. Considering the degree of connectivity as more than 1 km^2^, 22 important ecological nodes were selected, which were distributed unevenly in the north and southwest of the basin.

### 4.4. Construction and Optimization of Ecological Security Patterns

**Figure 6:**
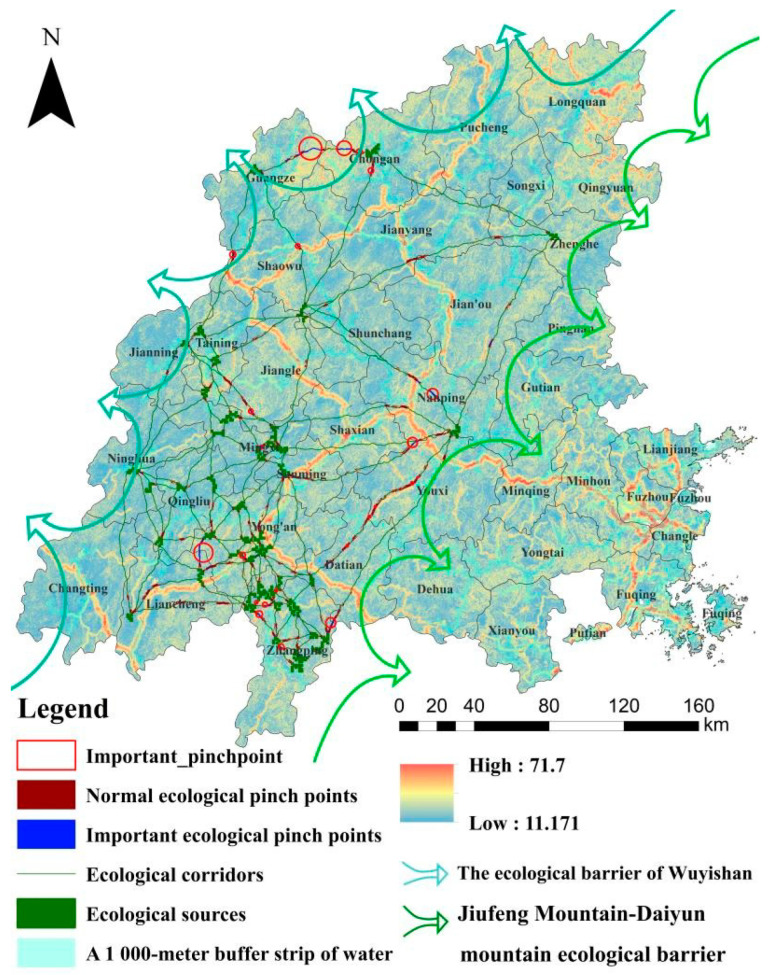
Construction of an ecological security pattern in the Minjiang River Basin.

Based on the ecological environment assessment and the MCR ecological resistance analysis, 43 ecological source areas, 118 ecological corridors, and 22 important ecological pinch points were integrated; finally, the ecological security pattern of “two screens, one area, multi-corridors and multi-nodes” in the Minjiang River Basin was constructed (Figure 6). The ecological optimization strategy was put forward according to the level of ecological security in the Minjiang River Basin. “Two screens” refer to the ecological barriers in Wuyishan and Jiufeng Mountain–Daiyun Mountain, which are located in the upper reaches of the Minjiang River Basin and the middle reaches of the Minjiang River Basin, respectively, and have the important functions of conserving water resources and protecting biodiversity; it is an important distribution area of subtropical evergreen broad-leaved forest in China and a key protected area of the watershed [43]. The “one belt” refers to the buffer zone of 1000 m around the main Minjiang River Basin and its main tributaries, which are relatively close to the Minjiang River Basin. It is necessary to control human disturbances to maintain water quality and protect biodiversity in the Minjiang River Basin.

The pattern of ecological security in the Minjiang River basin needs to be improved, mainly as follows: (1) Although there are many source areas, the spatial distribution is uneven, gathers in the southwest, and lacks contact with the north and east basins. The ecological corridor in the southwest is dense, the northeast side is singular and sparse, and it is easy to produce an ecological fracture. (2) The overall level of ecological security is relatively high, but local problems are prominent (Figure 6). The ecological development areas composed of high ecological security areas account for 65.8% of the total area of the watershed and are evenly distributed in space; almost all ecological sources and corridors are distributed in the high ecological security zone. This region is the main human activity and urban development area in the Minjiang River Basin, with low elevation, a gentle slope, a stable ecosystem, and strong anti-human-disturbance ability. The development and construction of this area should follow the principle of attaching equal importance to ecological protection and rational development and utilization [51]. The ecological control area, composed of middle and higher ecological safety areas, accounts for about one-third of the total area, about 27.7%. The area is mainly a low hill and gentle slope zone with a transition from mountain to basin; it is the edge area where the human activity space transforms into natural space. Because of the low altitude and gentle slope, the region not only provides a buffer for ecological space protection but also provides reserve space resources for social and economic development in hilly and mountainous areas. The ecological protection area is composed of the low ecological safety area, and the lower ecological safety area accounts for 6.5% of the total area and is concentrated in areas where human activities are more intensive. The area is strongly disturbed by human activities and has high ecological resistance; species connectivity is low. Conservation principles should be upheld, and large-scale human activities are not appropriate [51].

## 5. Discussion

The rapid development of a city leads to the deterioration of the ecological environment to a certain extent, which has a serious impact on the structure and function of the ecosystem itself, thus leading to the reduction of ecological resources and the weakening of the ability for socially sustainable development. In turn, the continued deterioration of the ecological environment will restrict economic development, resulting in a lack of government funds to protect forests, grasslands, water, and biodiversity. The Minjiang River Basin is one of the first pilot areas for ecological conservation and the restoration of mountain–river–forest–farmland–lake–grass in China. It plays an extremely important role in ensuring ecological security throughout the country and the province. However, in recent years, the basin has suffered serious ecological damage, such as soil erosion and biodiversity degradation [32].

At present, research on the eco-environmental problems of the Minjiang River Basin in China focuses on the spatiotemporal changes of a single feature [52,53], the assessment of ecological value [33], the quality of habitats, and so on [29,54]. There is little research on using quantitative assessment methods to construct the ESP of the Minjiang River Basin. For example, Ying [40] used qualitative methods to construct the ecological security pattern of the river basin in 2021. The construction of an ESP has been considered the basic guarantee for coordinating ecosystem protection and economic development in China, the components of which were identified in a bottom-line approach to protecting priority areas and controlling urban expansion [16,55]. The construction of an ESP in the Minjiang River Basin can not only identify its key protected areas but also explore its ecological background. It can also provide some reference basis for the construction of ESP in other areas of mountain–river–forest–farmland–lake–grass in China. Therefore, this study used a quantitative method to construct the ESP of the watershed, referring to the “Technical guide for evaluating the carrying capacity of resources and environment and the suitability of land and space development” issued by the Ministry of Natural Resources.

We carried out the research in the order of “identified the source, constructed the resistance surface, extracted the corridors and nodes, constructed and optimized the ESP.” It has gradually become the basic paradigm of ecological security pattern research. However, there has not been a unified standard for the extraction of ecological source areas; in the past, scholars have extracted them according to ecological sensitivity [18,56], biodiversity maintenance function importance evaluation [5], habitat quality evaluation [55], etc. To study the ecological and environmental problems in the Minjiang River Basin, and to identify areas with poor ecological environments, this paper evaluated the eco-environment of the watershed based on the ecological sensitivity and the importance of ecosystem function, which makes up for the lack of a single extraction ecological source.

There is growing evidence showing that construction activities usually occupy fertile farmland or forestry for construction activities, leading to urban expansion and the reduction of ecological land [57]. Our findings are consistent with several similar ESP studies [22,57]. In the identification of ecological source land, we found that some construction land was identified as an ecological source; the proportion of cultivated land and construction land in ecological source land was 15.87% and 1.91%, respectively, which reflects the contradiction between ecological protection and construction activities and agricultural production. In the process of constructing the ESP, we identified the areas with high ecological sensitivity and important ecosystem service functions to ensure the integrity and connectivity of the ecosystem. We explored the carrying capacity of resources and the environment of land space, identified the risk areas of land space development and utilization, and took protection measures for risk areas. We also found the most suitable space for agricultural production and urban construction.

Although this study is of great significance for improving the quality of the ecological environment and realizing regional sustainable development, the limitations of this study should also be noted. In the construction of the minimum cumulative resistance surface, because of the availability of data, this study considered both human factors, such as land use type, vegetation coverage, slope, and natural factors, such as distance from county roads and highways, which was more scientific. However, ecological corridors are not only affected by land use types, natural environments, or human disturbances. Other factors also play important roles in the choice of species migration pathways, such as the distribution of target species and the different abilities of species to search for resources. In particular, the attraction of enemies and escape from enemies may also affect the choice of species migration pathways [58]. Therefore, we hope that more factors can be taken intoaccount in the construction of the ESP in the Minjiang River Basin. In addition, due to the limitation of available data, the minimum scale of raster data used in this study is 1 km, which we hope to make up in the future.

## 6. Conclusions

Taking the Minjiang River Basin as an example, this paper evaluated its ecological environment by integrating the ecological sensitivity and the importance of the ecosystem functions to identify the ecological sources and combine the ecological corridors, the ecological nodes, the ecological barrier of Wuyishan, the ecological barrier of Jiufeng Mountain-Daiyun Mountain, and the 1000 m buffer zone for the main basin and tributaries of the Minjiang River Basin. The ecological security pattern of “two barriers, one belt, many corridors, and many spots” was constructed; the ecological protection area, the ecological control area, and the ecological development area were divided, and an ecological optimization strategy was put forward.

(1) The ecological security network has poor connectivity and low coverage, which only covers the middle and upper reaches of the Minjiang River Basin. The total length of the ecological corridor is 3,732,051.88 km; its shape is similar to a fishing net. The southwest side is dense, and it connects each important ecological source well, but the northeast side of the ecological corridor is singular, slender, and easy for an ecological fracture to occur. The ecological source land area is 523 km^2^, accounting for 0.6%, which is mainly forested land and cultivated land distributed in the southwest and northwest of the Minjiang River Basin, such as Zhangping. There are 22 important ecological pinch points unevenly distributed in the north and southwest of the basin. There are no ecological sources or corridors in the lower reaches of the basin.
(2) The overall level of ecological security is high, but some problems are prominent. The area with a high level of ecological security accounts for 65.8% of the total land area, and its spatial distribution is relatively uniform. The higher and medium ecological security zones, which account for 27.7% of the country’s land area, are mainly located in the Wuyishan and Jiufeng-Daiyun Mountains. The low ecological safety zone and lower ecological safety zone account for 6.5%, the ecological resistance value is high, the species connectivity is low, and we should adhere to the protection principle; the area is not suitable for large-scale human activities.

## Figures and Tables

**Table 1:** Classification and assignment of eco-environmental assessment factors in the Minjiang River Basin.

Factors	Low Sensitivity	Lower Sensitivity	Medium Sensitivity	Higher Sensitivity	High Sensitivity
*R*	<532	532–560	560–583	583–604	>604
*LS*	<70	70–123	123–178	178–248	>248
*C*	>0.95	0.85–0.95	0.75–0.85	0.55–0.75	<0.55
Soil texture	Silty soil	Sandy loam, loamy sandy soil, clay loam, silt (sand) clay loam, silt (sand) loam	Sandpaper clay, loam, (Sandy) loam	Sand, clay	Gravel
*I*	<0.55	0.55–0.65	0.65–0.75	0.75–0.85	>0.85
*W*	<100	100–140	140–190	190–250	>250
Hierarchical assignment	1	3	5	7	9

**Table 2:** Factor grade and weight in the ecological resistance analysis.

	Resistance Factor	Weight	Resistance Grade	Resistance Value
Natural disturbance	Land use type (C1)	0.095	Forest and grassland	20
			Water	40
			Cultivated land	60
			Bare land	80
			Construction land	100
	Vegetation coverage/(%) (C2)	0.213	>0.65	20
			0.50–0.65	40
			0.35–0.50	60
			0.15–0.35	80
			<0.15	100
	Slope/(%) (C3)	0.236	<5°	20
			5–15°	40
			15–25°	60
			25–35°	80
			>35°	100
Human disturbance	Distance from county road/m (C4)	0.118	0–150	20
			150–300	40
			300–450	60
			450–600	80
			600–800	100
			>800	1
	Distance from highway/m (C5)	0.173	0–400	20
			400–800	40
			800–1200	60
			1200–1600	80
			1600–2000	100
			>2000	1

**Table 3:** Pairwise comparison matrix.

	C1	C2	C3	C4	C5
C1	1	1/5	1/6	1/3	1/4
C2	5	1	1/2	4	3
C3	6	2	1	3	2
C4	3	1/4	1/3	1	1/2
C5	4	1/3	1/2	2	1

$\lambda_{max}$ = 5.1883; *CI* = 0.047; *CR* = 0.042 < 0.1.

**Table 4:** Area statistics of eco-environmental assessment factors in the Minjiang River Basin.

	Generally Important/Low Sensitivity/(%)	Relatively Important/Lower Sensitivity/(%)	Moderately Important/Medium Sensitivity/(%)	Highly Important/Higher Sensitivity/(%)	Extremely Important/High Sensitivity/(%)
*SSWL*	10.3	30.8	33.4	20.7	4.7
*SSE*	0.2	5.9	32.0	49.5	12.3
*SLD*	28.9	37.9	22.6	9.5	1.1
*WR*	17.3	29.3	26.1	19.0	8.4
*Spro*	72.8	19.3	7.5	0.2	0.1
*Sbio*	20.9	31.2	23.2	16.7	7.9
Comprehensive assessment of the ecological sensitivity	13.1	19.7	33.7	18.9	14.6
Comprehensive assessment of the importance of ecosystem function	33.7	28.8	14.8	14.1	8.6
Comprehensive assessment of the ecological environment	47.7	12.3	19.0	16.2	4.8

**Table 5:** Statistics of land use area in ecological source area.

Land Use	Area/(km^2^)	Proportion/(%)
Cultivated land	83	15.87
Forest and grassland	428	81.83
Water	2	0.38
Construction land	10	1.91

**Table 6:** Area statistics of ecological resistance factors.

Resistance Factor	Resistance Grade	Proportion of Area/(%)
Land use type	Forest and grassland	80.13
	Water	1.41
	Cultivated land	2.85
	Bare land	0.11
	Construction land	2.85
Vegetation coverage/(%)	>0.65	96.99
	0.50–0.65	1.75
	0.35–0.50	0.89
	0.15–0.35	0.34
	<0.15	0.04
Slope/(%)	<5°	0.12
	5–15°	0.38
	15–25°	0.33
	25–35°	0.14
	>35°	0.03
Distance from county road/m	0–150	1.58
	150–300	1.20
	300–450	1.21
	450–600	1.25
	600–800	1.35
	>800	93.41
Distance from highway/m	0–400	1.32
	400–800	1.34
	800–1200	1.37
	1200–1600	1.40
	1600–2000	1.47
	>2000	93.10

**Table 7:** Area statistics of Ecological Safety Zone classification.

Ecological Safety Zone	Area/(km^2^)	Proportion/(%)
High ecological safety zone	53,288.148	65.77
Higher ecological safety zone	16,858.311 km	20.81
Medium ecological safety zone	5608.967	6.92
Lower ecological safety zone	2269.87	2.80
Low ecological safety zone	3000.19	3.70

## Data Availability

The data presented in this study are available on request from the author. The data are not publicly available due to privacy. Images employed for the study will be available online for readers.
